# The Technology Dependence Index (TDI): Development and Validation in Neonatal and Pediatric Populations

**DOI:** 10.1002/nur.70121

**Published:** 2026-08-15

**Authors:** Ashlee J. Vance, Matthew Peterson, Hayley H. Estrem, Christine A. Fortney, Sharron L. Docherty, Debra H. Brandon

**Affiliations:** ^1^ Center for Healthcare Improvement Henry Ford Health Detroit Michigan USA; ^2^ College of Nursing Michigan State University East Lansing Michigan USA; ^3^ Department of Pediatrics, College of Human Medicine Michigan State University East Lansing Michigan USA; ^4^ School of Nursing The University of North Carolina at Wilmington Wilmington North Carolina USA; ^5^ College of Nursing The Ohio State University Columbus Ohio USA; ^6^ Center for Biobehavioral Health Nationwide Children's Hospital Research Institute Columbus Ohio USA; ^7^ Division of Nursing Science and Family Centered Health, School of Nursing Duke University Durham North Carolina USA; ^8^ Department of Pediatrics, School of Medicine Duke University Durham North Carolina USA

**Keywords:** caregiving complexity, psychometrics, technology dependence

## Abstract

The survival of infants and children with complex medical conditions continues to increase, underscoring the need to quantify technology dependence more effectively. Existing measures emphasize mortality risk and fail to capture the burden of ongoing technological support. To address this gap, the Technology Dependence Index (TDI) was developed and initially validated as a standardized measure capturing the technology‐dependence component of neonatal and pediatric caregiving complexity. Technology dependence is defined as the complexity and intensity of the skilled, health‐related caregiving an infant or child needs to sustain physiological functioning, beyond the presence of any single device. Pooled data from six studies yielded 818 TDI observations across 265 infants. Inter‐rater reliability was moderate (*κ* = 0.61), with intraclass correlations from 0.90 to 0.99 and at least 90% agreement for 10 of 12 domains, noting the medication domain had the lowest inter‐rater reliability (45%–48%). Principal components analysis supported a multidimensional internal structure, with the 12 domains loading onto three retained factors. Researchers and clinicians can use the TDI retrospectively through chart review, prospectively in real time, or via parent report, making it adaptable to both research and clinical settings.

## 1 Introduction

Advances in obstetric, neonatal, and pediatric care have increased the survival rates of infants and children with complex chronic conditions (i.e., prematurity, congenital heart disease, cancer, and brain injuries), yet many of these children continue to have significant medical complexity including impairments associated with illnesses or side effects of treatments (Mahon 2004; Rogers et al. 2021; Yu et al. 2022). Many infants and children experience additional physical, functional, or mental impairments, further exacerbating their primary medical diagnosis; thus, increasing the level of caregiving complexity.

Children with complex medical conditions require coordination of multiple healthcare‐related services to achieve optimal outcomes compared to children without health problems; this constellation of caregiving issues is often referred to as medical complexity (Cohen et al. 2011; de Banate et al. 2019; Murphy et al. 2025). Approximately 13% of children in the United States have special healthcare needs, including specialized physical, occupational, or speech therapies, specialized medical treatments, prescription medications, and assistive technologies (Heneghan et al. 2022; Yu et al. 2021). Children with complex medical conditions have higher healthcare utilization, including greater prescription use, more outpatient visits and procedures, more frequent emergency or urgent care, and are more likely to be technology dependent (Hannan et al. 2021; Houtrow et al. 2011; Yu et al. 2021). Caregiving complexity is defined as the effort put forth by a caregiver exceeding that of routine caregiving or parenting activities for a healthy child, and often includes management of complex medical technology, addressing functional limitations requiring external assistance, and care coordination for medical care, and extends to psychosocial needs (i.e., financial or social support) (Rogers et al. 2021). Technology dependence refers to infants or children with chronic conditions, often with acute exacerbations, who require technological support, such as mechanical ventilation, tracheostomy, medications, feeding tubes, or oxygen delivery, provided continuously or intermittently throughout the day to sustain life, either in the hospital or home setting (Spratling 2015). Despite this broad definition, researchers and clinicians lack a standardized tool to quantify the degree and intensity of technology dependence and the associated caregiving complexity that families experience.

Existing severity‐of‐illness measures capture the frequency of specialized healthcare services, therapies, medications, and technological assistance that critically ill infants and children require, but each was designed to predict a specific outcome within a fixed window of care: the Nursey Neurobiologic Risk Score (NBRS) (Brazy et al. 1991), predicts neurodevelopmental impairment from acute insults during the early NICU course in preterm infants; the Score for Neonatal Acute Physiology (SNAP) (Richardson et al. 1993) and SNAP‐II/SNAPPE‐II (Richardson et al. 2001) predicts neonatal mortality from physiologic derangement in the first 12–24 h after admission; and the Pediatric Risk of Mortality score (Pollack et al. 1996) predicts in‐hospital mortality from physiologic instability during the initial PICU period. Across these instruments, the unifying construct is risk of death or impairment, derived from illness severity, and each is validated for a fixed‐time event (e.g., admission), setting (e.g., intensive care), or population (e.g., preterm infants). None characterizes the sustained, evolving burden of skilled caregiving and technological support a child requires across the clinical trajectory, including after discharge, nor yields a repeated, within‐patient measure of technology dependence over time (Grunberg et al. 2019; Marcin and Pollack 2000), This gap, availability of an instrument capturing technology dependence as a component of caregiving complexity and its change over time rather than one‐time mortality or impairment risk, was the rationale for the development and use the Technology Dependence Index (TDI).

### 1.1 Measure Development

#### 1.1.1 Initial Development

The TDI, formerly named Technology Dependence Scale, was developed and refined across multiple study phases (Figure 1). The measure, Technology Dependence Scale, was first developed for use in a large observational R01 study of 105 infants with serious life‐threatening health problems due to prematurity, birth defects, or chronic disease, to determine how technology dependence during hospitalization of medically fragile infants impacted parental role attainment (Docherty et al. 2002; Lee et al. 2007; M. Miles 1998; M. S. Miles et al. 1999). The measure was developed to address limitations in other illness severity measures and to provide a tool for quantifying the degree of technology dependence, a component of neonatal and pediatric caregiving complexity.

**Figure 1:**
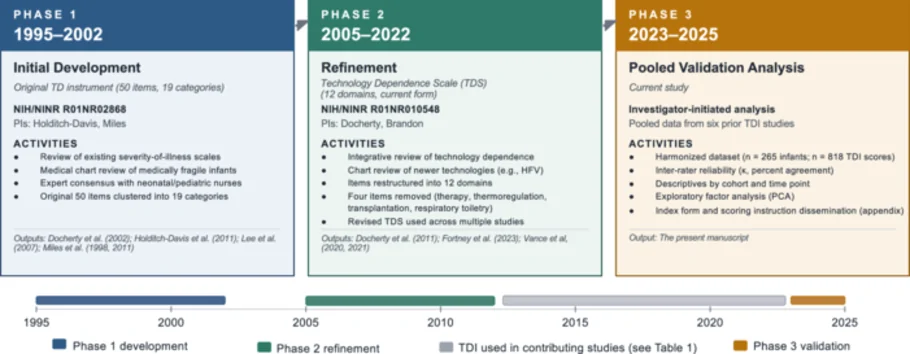
Timeline of the technology dependence index (TDI).

To create the measure, the study team began with a review of existing severity of illness scales (Horn and Horn 1986; Richardson et al. 1993; Yeh et al. 1984), a systematic review of empirical literature (unpublished work), medical chart reviews, and expert consensus with experienced neonatal and pediatric nurses. This review of empirical literature evaluated how the severity of illness was described across different patient populations, settings, and diagnostic categories and determined that existing scales did not capture factors related to technology dependence. Medical charts were reviewed for those with medical complexity to develop scale items: mechanical ventilators, prolonged intravenous administration of nutritional substances or drugs, device‐based respirator or nutritional support including tracheotomy tube care, suctioning, oxygen support, or tube feeding, and medical devices that compensate for vital body functions requiring daily or near‐daily nursing care. The first version of the measure included 50 items, clustered into 19 categories based on the type of technology (e.g., parenteral infusion lines, monitoring equipment, oxygen monitoring, monitoring lines, neurologic devices and monitors, oxygen, respiratory dependence, tracheostomy, respiratory toiletry, gastrointestinal feeding equipment, total parenteral nutrition, medications, transfusions, chest tube, elimination technology, therapy, and transplantation).

#### 1.1.2 Refinement

After the R01 study, it was determined that the measure needed refinement to include changes in how systems cared for infants and children with medical complexity, such as those discharged from the hospital but with ongoing healthcare needs requiring specialized care in the home. An updated review of empirical literature was needed to determine how technology dependence is measured across different patient populations, settings, and diagnostic categories. Results from this integrated review demonstrated that technology dependence was a part of the larger concept of illness severity with four interrelated dimensions of severity of illness: (1) degree of derangement of physiologic variables, (2) complications associated with the illness, (3) stage of the illness, and (4) level or degree of technological support (Docherty et al. 2011). Medical chart review indicated that a modification of the original items from the TD was needed, and new items were added to capture the concept of severity of illness. For example, infants and children with derangement of physiologic variables required multiple physiologic and biochemical assessments either in the hospital or at home. Complications associated with the illness or treatments were described as increased monitoring of skin integrity and treatments to maintain skin integrity and oral hygiene. The stage of the illness was represented by the additional types of monitoring the infant or child required, the types and routes of medications prescribed, and the general location where the infant or child was receiving care. Finally, the technological devices used were updated to include newer types of technology (i.e., high‐frequency ventilation).

After generating new items, the investigators revised the measure into 12 categories, then known as the Technology Dependence Scale: (1) care environment, (2) monitors, (3) respiratory assistance, (4) invasive lines, (5) external drains and/or catheters, (6) skin care, (7) oral care, (8) nutrition, (9) blood draws, (10) colloid therapy, (11) mobility, and (12) medications. Four items were eliminated from the original TD measure, which included therapy (i.e., occupational and physical therapy), thermoregulation, transplantation, and respiratory toiletry. Therapy was eliminated because infants and children using assistive devices for mobility generally receive therapy. Thermoregulation and transplantation were eliminated because they were illness‐specific, and respiratory toiletry was removed since those receiving respiratory support were provided with respiratory toiletry as part of their standard care. Therefore, the revised measure included 12 categories addressing the components of illness severity in infants and children.

The revised measure has been used in multiple studies of infants admitted to the Neonatal Intensive Care Unit since 2011. In each of these studies, technology dependence was defined as the complexity and intensity of the health‐related caregiving an infant or child requires to sustain health, growth, and physiological functioning. It reflects the volume, frequency, and skill level of the physical caregiving tasks. For example, airway and breathing support, enteral or intravenous feeding, medication administration, physiologic monitoring, and skin, line, drain, and elimination care, that must be performed beyond the ordinary care of a healthy child of the same age. Although the medical devices and equipment a child uses often drive these demands, the concept is not limited to the presence of equipment; it encompasses the hands‐on administration, monitoring, and management of medical care that the child's condition requires. Preliminary findings suggest that lower technology dependence scores (i.e., lower dependence/illness severity) were associated with greater maternal psychological well‐being (Vance et al. 2020) and increasing parenting confidence over time (Vance, Costa, et al. 2021). Conversely, there is evidence that greater technology dependence was the strongest predictor of maternal caregiving stress (Docherty et al. 2002, 2007). Thus, the TDI quantifies the technology‐dependence component of caregiving complexity by measuring the level of technological support required by an infant or child.

Technology dependence represents a critical component of caregiving complexity, influencing parenting and family health by assessing the demands of complex medical care. Because the field lacked a standardized, validated means of quantifying technology dependence, an empirically based, rigorously developed measure such as the TDI is essential to operationalizing this construct and to advance understanding of its impact on infants and families alike. Conceptually, the TDI is anchored in the illness‐severity framework described by Docherty et al. (2011), in which technology dependence represents one component of caregiving complexity from which the original TD items were generated. The present study was guided by an instrument‐validation framework grounded in classical test theory and contemporary standards for evaluating health measurement instruments. Building on this foundation, the purposes of this study were twofold: (1) to describe the development and psychometric validation of the TDI, including its internal structure and feasibility across multiple modes of data collection, and (2) to examine how TDI scores vary across clinical populations and care‐setting time points using pooled study data.

## 2 Methods

### 2.1 TDI Validation

This present study aimed to validate the use of the TDI and examine the range of scores across neonatal and pediatric populations. As described above, the measure was developed across phases: Phase 1 (1995–2002), initial measure development for use in an R01 study; Phase 2 (2005–2022), measure refinement and use across multiple studies; and Phase 3 (current study), measure validation and dissemination (Figure 1). The validation analysis for the TDI utilized data pooled from six studies, which are described in Table 1. Each study used the TDI as a repeated measure at the level of the individual infant: the same infant was scored at multiple points along their own clinical trajectory rather than different infants being sampled at successive waves. Within a given infant, the longitudinal scores reflect that same infant's changing technology dependence over time. These time points spanned birth or hospital admission, the inpatient period (during hospitalization and/or near discharge), and the post‐discharge (outpatient) period.

**Table 1:** Study characteristics of pooled data.

	Design	Purpose	Setting	Participants	TDI data collection
Study 1: Maternal caregiving	Observational	To examine responses and long‐term adjustment of mothers providing acute care to their children post‐hematopoietic stem cell transplant (HSCT).	BMTU	62 pediatric bone‐marrow transplant patients (< 18 years old)	1. 7 days post‐transplant 2. Discharge 3. 90‐days post‐transplant 4. 180‐days post‐transplant
Study 2: Cycled light	Randomized controlled trial	To test whether a cycled light intervention, in which preterm infants received varying amount of day and nighttime light, to determine if light impacted developmental outcomes.	NICU	20 infants born preterm (< 31 weeks GA)	1. Birth/admission 2. 7 days after admission 3. Discharge 4. 9 months after discharge
Study 3: Decision making	Mixed‐methods	To examine the trajectory of decision making for infants with complex life‐threatening conditions and influences on parents’ decision making.	NICU, PCICU	33 infants with complex life‐threatening conditions & their caregivers	1. Birth/admission 2. Weekly after admission 3. Monthly after discharge
Study 4: Parent confidence	Longitudinal survey	To describe the development of parent confidence over time among parents of infants with medical complexity.	NICU, PCICU	67 infants born preterm (< 37 weeks GA) and with medically complex conditions	1. Birth/admission. 2. Weekly after admission 3. Discharge 4. Post‐discharge (3 months)
Study 5: Bundled care	Observational	Explore the impact of bundling nursing care activities on the overall health of VLBW preterm infants in a Level IV NICU.	NICU	46 infants (28–32 weeks GA)	1. Weekly, once stable (e.g., full‐feeds, Q3 NICU cares)
Study 6: Infant symptoms	Longitudinal survey	To characterize the symptom experience of critically ill infants at high risk of dying.	NICU	78 infants (23–41 weeks GA) born with medical complex conditions	1. Birth/admission 2. Weekly after admission 3. Discharge

Abbreviations: BMTU, bone marrow transplant unit; GA, gestational age; NICU, neonatal intensive care unit; PCICU, pediatric cardiac intensive care unit; TD, technology dependence.

### 2.2 TDI Scoring

The TDI can be scored by a trained study team member (e.g., research assistant, nurse, graduate student), using data from a medical chart review for hospitalized patients or from parental reports or chart review after discharge. The scoring of the TDI is cumulative, with each point increment indicating a greater level of dependence or complexity across the 12 domains. The response options are either single‐select (radio buttons) or multiple‐select options (checkboxes). Each domain was designed with 1‐point differences reflecting an increase in technology dependence, except for the medication domain. Scoring for the medication domain assesses not only the number of medications, but also the frequency of administration and the route of medication. For example, medications have point values associated with the route and frequency of administration. The conceptual rationale for the incremental scoring of medications considers that medication administration is nonlinear, meaning there is not a 1:1 alignment with the number of medications administered. Caregivers and nurses often administer multiple medications in a single routine, so the marginal burden of each additional medication is smaller than adding a new device or technology. Thus, the range of possible scores on the TDI is 0 to ∝ (i.e., no ceiling) with higher scores indicating more dependence and thus greater caregiving complexity. The TDI form and scoring instructions are available as supplemental content.

### 2.3 Pooled Data Set and Analysis

Each study was approved as human subjects’ research by the respective authors’ Institutional Review Board. Research assistants for each study reviewed infant demographic data and scored the TDI from a medical chart review during the inpatient hospitalization and via parent report and medical chart review for outpatient scoring. Because the six studies were designed independently, their data‐collection schedules differed in both timing and frequency (Table 1). To balance and pool the data, two members of the research team (D.H.B. and A.J.V.) independently reviewed each study's original collection time points and mapped them onto a common ordinal time framework representing consistent phases of care during and after hospitalization. Four ordinal categories were defined so that each value carried the same meaning across all studies: (1) admission, the first TDI score recorded for a participant (i.e., at birth); (2) hospitalization, scores obtained during the hospital stay, of which a participant could contribute one or more scores, designated 2.1, 2.2, 2.3, and so on when multiple inpatient scores were recorded; (3) discharge, a score obtained near or on the day of discharge, or the last score recorded during hospitalization; and (4) outpatient, a score obtained 3−9 months after discharge. By design, not all studies contributed scores at every time point; these structural absences reflect each study's original protocol rather than missing data. The study population was harmonized into a common variable (i.e., preterm, medically complex/cardiac, and bone marrow transplant unit) to support comparison across cohorts. The analytic data set was de‐identified for analysis, removing all dates and other identifiers.

We conducted a complete‐case analysis: no values were imputed, and only participants with complete TDI scores were retained in the pooled data set. Descriptive statistics were computed on demographic data for each study. Reliability, validity, and descriptive statistics of TDI scores were analyzed using the pooled data set. To evaluate scoring consistency, we conducted inter‐rater reliability using kappa values and percentage of agreement, calculated as the difference in TDI scores between the two raters. Test−retest reliability was not evaluated because the level of technology dependence of an infant or child is not expected to remain constant over time. An exploratory factor analysis was conducted on the pooled baseline data to examine structural validity. The TDI comprises 12 domains, each scored as a single select option or multiple select for a cumulative score in that domain. Dimensionality was examined using principal components analysis based on a Spearman's rank‐order correlation matrix. Spearman's correlations were selected as a distribution‐free index of monotonic association appropriate for items measured on heterogeneous, non‐interval scales. Data were analyzed using SAS statistical software (SAS, Cary, NC).

## 3 Results

Infant demographic characteristics for each study are presented in Table 2. There were 265 infants and 818 TDI observations across the six studies. Descriptive statistics for the TDI scores by study are provided in Table 3 and TDI scores by study population in Table 4. Studies 1 and 2 had data from two raters to assess inter‐rater reliability. The kappa value for inter‐rater reliability was 0.61. Ten of 12 items had > 90% agreement in rater scores at time 1, and agreement on medications varied from 45% to 48% agreement across each time point (Supporting Information S1: Table S1). Intraclass correlations ranged from 0.90 to 0.99 for TDI scores between study 1 and study 2 (Supporting Information S1: Table S2).

**Table 2:** Infant demographics from the six studies contributing data.

	Study 1	Study 2	Study 3	Study 4	Study 5	Study 6
	Maternal caregiving (*N* = 20)	Cycled light (*N* = 20)	Decision making (*N* = 33)	Parenting confidence (*N* = 67)	Bundled care (*N* = 46)	Infant symptoms (*N* = 79)
Gestational age (weeks) (M, SD)	na	26 (1.6)	52 (33.1)	33 (5.7)	28 (2.8)	33 (5.4)
Sex, male (*N*, %)	na	12 (60%)	11 (33%)	37 (56%)	21 (46%)	46 (58%)
Length of stay (days) (M, SD)	na	122 (42)	na	63 (45)	na	60 (43)^a^
Race/ethnicity (*n*, %)						
White	na	7 (35%)	15 (46%)	42 (63%)	21 (46%)	48 (61%)
Black	na	13 (65%)	10 (30%)	13 (19%)	14 (30%)	17 (22%)
Asian	na	0	0	0	2 (4%)	0
Other	na	0	6 (18%)^b^	6 (9%)^c^	4 (9%)^d^	3 (3%)^e^
Hispanic	na	0	2 (6%)	6 (9%)	5 (11%)	11 (14%)
Study population (*N*, %)						
Preterm (*n* = 124)	0	20 (100%)	10 (30%)	18 (27%)	46 (100%)	30 (38%)
Medically complex (*n* = 110)	0	0	12 (36%)	49 (73%)	0	49 (62%)
BMTU (*n* = 31)	20 (100%)	0	11 (33%)	0	0	0

Abbreviations: BMTU, bone marrow transplant; N, sample size of infants enrolled in the study; na, information not available or collected for this study.

^a^Calculated as the time enrolled in the study, ^b^Includes American Indian and Other, ^c^Includes American Indian, Native Hawaiian or other Pacific Islander, and more than one race, ^d^Includes American Indian, Native Hawaiian or other Pacific Islander, and other or more than one race, ^e^Includes American Indian, Native Hawaiian or other Pacific Islander.

**Table 3:** TDI Scores by study using balanced time points.

	T1‐admission		T2‐inpatient		T3‐discharge		T4‐outpatient	
	M (SD)	Min/Max	M (SD)	Min/Max	M (SD)	Min/Max	M (SD)	Min/Max
Study 1: Maternal caregiving (*n* = 118)	41.1 (5.6)	33.5/52.2	—	—	26.9 (5.0)	19.7/36.7	25.5 (13)	10.2/60.7
Study 2: Cycled light (*n* = 120)	25.6 (5.0)	17/38	29.6 (6.8)	20.7/48.0	30.7 (8.1)	17/47.5	—	—
Study 3: Decision making (*n* = 81)	30.3 (8.6)	16.7/57.2	37.8 (11.8)	16.75/57.25	36.4 (27)	1.75/130.75	16.8 (13.6)	1/54.25
Study 4: Parent confidence (*n* = 192)	23.5 (6.4)	11/43	21.7 (16.1)	7.5/ 41.5	16.1 (7.4)	7.5/ 43.5	6.8 (6.7)	1/ 41.5
Study 5: Bundled care (*n* = 158)	14.8 (2.9)	8.7/ 21.5	14.4 (3.2)	9.5/ 24.5	13.2 (3.8)	7/ 24	—	—
Study 6: Infant symptoms (*n* = 149)	24.9 (11.1)	10/ 72	22.6 (8.8)	9/ 55.75	15.1 (6.0)	7/ 31.5	—	—

Abbreviation: *n*, number of TDS observations.

**Table 4:** TDI Scores by population using balanced time points.

	T1‐admission		T2‐inpatient		T3‐discharge		T4‐outpatient	
	M (SD)	Min/Max	M (SD)	Min/Max	M (SD)	Min/Max	M (SD)	Min/Max
Preterm (*n* = 509)	23 (8.9)	8.772.5	21.7 (8.8)	955.75	18.4 (9.7)	1.748	4.7 (2.9)	112.25
Medically complex (*n* = 157)	24.8 (9.4)	1157.2	25.1 (13.5)	7.567	21.2 (19.1)	7.5130	9.6 (10.8)	154.25
Bone marrow transplant (*n* = 152)	37.7 (8.4)	16.752.2	34.1 (8.5)	16.7543.75	30.3 (12.3)	19.793	24.4 (12.5)	10.260.75

Abbreviation: *n*, number of TDS observations.

Principal components analysis was conducted to test the internal structure validity, since the items were generally correlated with each other. From the 12 items, three factors were retained in the final rotated model, and presented in Table 5. One item (skin care) loaded onto factors 1 and 3 with similar values (0.41, 0.35, respectively). Two items (nutrition and external drains) had negative factor loadings, indicating an inverse relationship with factor 2. The last item on the measure (mobility) did not load onto any factor because it had no variance given that all scores for this item were zero across all studies. However, it has been retained in the Index as it is a relevant domain for older pediatric patients who may have been born preterm or with medically complex conditions.

**Table 5:** Exploratory factor analysis.

	Factor loadings (% variance)		
	1 (2.29)	2 (2.16)	3 (1.92)
*Factor 1—Stage of illness*			
Care environment	0.61		
Parenteral lines	0.77		
Respiratory assistance	0.85		
Skin care	0.41		
*Factor 2—Physiologic needs*			
Monitors		0.56	
Oral care		0.72	
External drains and catheters		−0.56	
Nutrition		−0.73	
*Factor 3—Procedures/complications*			
Blood draws			0.51
Colloid therapy			0.75
Medication			0.84
Mobility			0

## 4 Discussion

This study demonstrates that the TDI is an appropriate tool for measuring the technology‐dependence component of neonatal and pediatric caregiving, a component that is robustly associated with, but does not by itself constitute, the full construct of caregiving complexity. In contrast to prior measures that emphasize mortality risk, the TDI captures the degree of technological support required by infants and children across diverse care settings, enabling more precise assessment of technology dependence, a component consistently associated with caregiver burden (Matsuzawa et al. 2022; Pitch et al. 2023; Shattnawi et al. 2023). Importantly, the TDI is a flexible tool that can capture technology dependence retrospectively (chart review), prospectively (in real time), or via parent report (outpatient/home), and was developed and refined using empirical data and applied across multiple studies, ensuring its relevance and utility in diverse pediatric populations (Fortney et al. 2023; Vance et al. 2020). This flexibility allows the index to be used in research to better understand the influence of technology dependence on infant and family outcomes and understand changes in routine care delivery; thus, relevant in both research and clinical practice.

A unique strength of the TDI is its ability to capture changes in technology dependence over time, allowing for longitudinal assessments. Unlike categorical measures restricted to single diseases or care contexts, the TDI uses a flexible scoring system that distinguishes between low and high levels of dependence across acute, critical, outpatient, and home environments. This granularity makes the tool particularly valuable for examining how caregiving demands evolve, identifying populations with persistent high dependence, and comparing patterns across diverse samples. Such applications may also reveal inequities in caregiving burden and access to resources, providing evidence that can guide interventions to improve care coordination and reduce disparities.

Two clarifications are warranted regarding the conceptual scope of the TDI. First, although the foundational definition of technology dependence in this work draws from Spratling 2015 integrative review, no consensus replacement definition has emerged. Recent literature suggests that technology dependence remains inconsistently defined, with most operationalizations relying on individual investigator opinion (Brenner et al. 2021; Zorko et al. 2023). We retained Spratling 2015 definition for the TDI because it is the only definition derived through systematic synthesis of the empirical literature and because it explicitly distinguishes the continuous‐versus‐intermittent and life‐sustaining elements that the TDI scoring structure was designed to quantify.

Second, we acknowledge that caregiving complexity is itself a multidimensional construct that encompasses not only technological tasks but also socioemotional, psychological, financial, and spiritual dimensions that the TDI does not directly measure. Social determinants of health are likewise increasingly recognized as critical, separate determinants of family well‐being in this population (Giambra and Spratling 2023). The TDI should therefore be understood as a standardized measure of one well‐evidenced component of neonatal and pediatric caregiving complexity rather than as a substitute for its broader assessment. Empirical work supports this positioning: in studies of children who require medical technology, higher technology dependence has been consistently associated with greater caregiver workload, sleep loss, financial strain, depressive symptoms, and reduced health‐related quality of life (Caicedo 2014; Lawrence et al. 2025; Pitch et al. 2023). Furthermore, authors’ prior work demonstrated that lower TDI scores are linked to greater maternal psychological well‐being and parenting confidence over time (Docherty et al. 2007; Vance, Costa, et al. 2021; Vance, Knafl, et al. 2021; Vance et al. 2020). Used alongside instruments that measure the psychosocial, economic, and contextual dimensions of caregiving, the TDI contributes a precise, replicable measurement of the technology‐dependence component of caregiving complexity that is otherwise inconsistently captured in clinical records and research. Future research is needed to better understand the association of technology dependence with other social factors and components of caregiving complexity.

The psychometric validation confirmed the reliability and validity of the TDI, and areas for refinement. Two items had negative factor loadings, nutrition and external drains, suggesting limited contribution to the overall construct or potential redundancy when single interventions (e.g., tube feeding) are captured in multiple categories. While the TDI effectively measures technology dependence, future refinements may be needed to address areas of overlap and ensure that item scoring accurately reflects the intended construct. This includes the mobility and medication domains. With the inter‐rater reliability being low for the medication domain, it is important to recognize that accurately documenting medications is a known challenge in complexity measurement (Condren et al. 2019). Medication lists are complex, inconsistently documented, and require interpretation, unlike discrete technologies (e.g., ventilator, G‑tube) that are binary and obvious. This is an important area for discussion when training a research team member to score the TDI, and we offer suggestions on how best to do this in the scoring instructions. As for the mobility item, which demonstrated no variance in this study (i.e., zero), it was retained in this analysis to preserve its broader applicability to pediatric populations in which mobility technologies or functional limitations are more prevalent. Thus, the mobility domain could be removed if not relevant to the study population and would not change the overall TDI score.

Although the TDI scoring process is structured and adaptable, deliberate training and ongoing verification are essential to preserve the integrity of the scores generated. Where feasible, we recommend recruiting study staff who are already familiar with medical records or who have a general understanding of clinical practice and workflow. Training should equip these staff to navigate the medical record, locate the required information across different electronic health record platforms (e.g., distinguishing inpatient from outpatient data), and understand each domain in sufficient depth to articulate, when necessary, what information must be obtained directly from parents. To establish inter‐rater reliability, we encourage a “see one, do one, teach one” approach paired with a structured dual‐verification process. For example, develop a plan for two team members to independently score every tenth participant or having the principal investigator, or a staff member most familiar with clinical data, review every twentieth score.

Because the instrument accommodates multiple data‐collection methods, including medical chart review and parent report, this training and verification structure can be applied consistently across both inpatient and outpatient settings. To support accurate abstraction, the scoring instructions also include workflow guidance for locating the relevant clinical information within electronic medical record systems such as EPIC. Several of the studies contributing to the pooled data used REDCap, a research data‐management platform, including its automated scoring calculations, to improve both the efficiency and accuracy of TDI scoring. For initial training, we recommend scoring on paper and confirming agreement among abstractors before entering data into a data set (e.g., Excel or REDCap), and research teams may find it useful to review TDI scores during team meetings to verify the underlying clinical information.

## 5 Limitations

While the TDI was used across multiple studies, populations, and settings, the samples were predominantly hospitalized infants. This emphasis reflects the population in which technology dependence is most visible and where validation was most feasible. However, caregiving complexity often evolves as infants transition into home and community environments, where families assume greater responsibility for managing technological supports. Expanding the application of the TDI to these settings would be critical for capturing the full continuum of caregiving demands. Another limitation of this validation study is the absence of a gold standard measure for technology dependence, which precluded direct assessment of convergent validity. Relatedly, because the TDI quantifies only the technology‐dependence component of caregiving, it should be paired with measures of the psychosocial, financial, and contextual dimensions of caregiving when the research question concerns caregiving complexity as a whole construct. Given its conceptual differences from illness severity indices, direct comparisons were not appropriate. Instead, validity was demonstrated through use across diverse populations and study designs, showing the capacity to capture variation in technology dependence. Lastly, a consistent and replicable process for reviewing medication documentation may improve inter‐rater agreement and enhance reliability.

## 6 Conclusion

The TDI represents a significant advancement in the measurement of the technology‐dependence component of neonatal and pediatric caregiving complexity, offering a standardized approach to quantifying technology dependence across different healthcare settings. The TDI can evaluate changes over time and compare caregiving demands across diverse populations, making it a valuable tool for both research and clinical practice. Beyond measurement, the TDI provides critical data that can inform interventions to improve healthcare delivery, strengthen caregiver supports, and optimize resource allocation. Continued application and refinement of the TDI will solidify its role in improving care and the management of complex caregiving needs, while future work linking child and family outcomes will ensure its relevance to holistic, family‐centered care.

## Author Contributions

**Ashlee J. Vance:** conceptualized the manuscript, assisted in data acquisition, oversaw analyses, contributed to interpretation of findings, and drafted and revised the manuscript. **Matthew Peterson:** provided expertise in psychometric methods, conducted analyses, contributed to the interpretation of findings, and critically reviewed the manuscript. **Hayley H. Estrem:** assisted in data acquisition, contributed to the interpretation of findings, reviewed, and critically revised the manuscript. **Christine A. Fortney:** assisted in data acquisition, reviewed, and critically revised the manuscript. **Sharron L. Docherty:** conceptualized the manuscript, led index development, assisted in data acquisition, contributed to interpretation of findings, and critically reviewed the manuscript. **Debra H. Brandon:** conceptualized the manuscript, led index development, assisted in data acquisition, contributed to interpretation of findings, and critically reviewed the manuscript.

## Conflicts of Interest

The authors declare no conflicts of interest.

## Supporting information


Supporting File

## Data Availability

The data that support the findings of this study are available from the corresponding author upon reasonable request.
