# Supplementary material for: The Technology Dependence Index (TDI): Development and Validation in Neonatal and Pediatric Populations
Source: Res Nurs Health. 2026 Aug 15;49(6):899–908. doi: 10.1002/nur.70121 (PMC13729730; doi:10.1002/nur.70121)
Supplement: Supplementary file 1 [file NUR-49-899-s001.pdf]

TECHNOLOGY DEPENDENCE INDEX

**1. Care Environment**<br>(Circle one)	Critical Care<br>(ICU)<br>(3)	Intermediate Care<br>(Step-down, Special care)<br>(2)		Outpatient<br>(Complex)<br>(1)	Outpatient<br>(Home)<br>(0)	
**2. Monitors**<br>(Check all that apply)	Pulse Ox<br>[1]	Cardiac – Respiratory<br>(ECG leads)<br>[1]		Blood<br>Pressure<br>[1]	Other<br>(Home monitor)<br>[1]	
**3. Respiratory Assistance**<br>(Check all that apply)	Room air<br>(no support)<br>[0]	Nasal<br>Cannula<br>(≥21% 02)<br>[1]	CPAP,<br>BIPAP,<br>RAM<br>(≥21% 02)<br>[2]	Trach*<br>[3]	Ventilator<br>(ETT)<br>[4]	HFV<br>or Jet<br>(ETT)<br>[5]
**4. Invasive Lines**<br>(Check all that apply; 1 point per line)	PAL<br>'A-line'<br>[1]	UAC<br>[1]	UVC<br>[1]	PICC<br>[1]	PIV<br>[1 per IV]	Central Line or<br>Other<br>[1 per line]
**5. External Drains and Catheters**<br>(Check all that apply; 1 point per line)	Feeding Tube<br>(OG/NG/GT)<br>[1]	Replogle<br>or similar<br>[1]	Chest<br>Tube(s)<br>[1]	Foley<br>[1]	Abdomen Drains<br>(penrose, etc)<br>[1 per tube]	
**6. Skin Care**<br>(Check all that apply)	Skin<br>Monitoring<br>Daily<br>[1]	Topical<br>Applications<br>[2]<br>Routine line<br>dressing<br>changes<br>[2]	Complex<br>dressing<br>changes<br>(surgical or<br>wound<br>care)<br>[3]	Ostomy<br>dressing &<br>maintenanc<br>e<br>[4]	Open surgical<br>wound<br>[5]	
**7. Oral Care**<br>(Circle one)	Routine<br>(0)		Intermediate<br>(1)		Complex<br>(2)	
**8. Nutrition**<br>(Check all that apply)	PO Intake<br>(Chest or Bottle)<br>[1]		Tube (Gavage) Feedings<br>(OG/NG/GT)<br>[2]		TPN<br>[3]	
**9. Blood Draws** _ _ _ _ _ _ _<br>(Circle one; count total in 7 days)	None<br>(0)	Once a week<br>(1)		2 – 6 draws<br>(2)	Daily<br>(3)	
**10. Colloid Therapy**<br>(Check all that apply; 1 point per transfusion)	Blood Products<br>[1]	Platelets<br>[1]		Albumin<br>[1]	Other<br>[1]	
**11. Mobility**<br>(Check all that apply; 1 point per device)	Special Car Seat<br>[1]		Walker<br>[1]	Wheelchair<br>[1]	Special Stroller<br>[1]	
**12. Medications**<br>(For **each** medication given: Circle point box and add up total score for all medications given on date of scoring)	**Frequency:**	**Daily**	**2-3x day**	**4-6x day**	**Continuous**	
	**Route:**<br>IV, IM, ET<br>Sub Q, Nebu	1	1.25	1.50	1.75	
	**Route:**<br>PO, Topical,<br>Rectal, IN	0.5	0.75	1	NA	

BIPAP: Bidirectional positive airway pressure, CPAP: Continuous positive airway pressure, ETT: Endotracheal tube, HFV: High frequency ventilation, IN: Inhalation IV: Intravenous, IM: Intramuscular, NC: Nasal canula, Neb: Nebulizer, Trach: Tracheostomy

TECHNOLOGY DEPENDENCE INDEX

Scoring Instructions

TDI provides a cumulative numerical value associated with technology dependence on the date of scoring. The TDS can be scored daily, weekly, monthly, or at significant transition points (e.g., change in level of care or discharge). Information for scoring can be based on a chart review or parent report. Scores from each domain are totaled and then summed for a total cumulative score.

To score the TDI, we have defined each domain, scoring options and additional instructions. We also provide (in bold) workflow options for locating information in an electronic database system (e.g., EPIC). To score using a REDCap form or other online database, *circle one* options should be formatted as radio buttons (single option) and *check all that apply* as check boxes (multi-select). For TDI scoring, the location of each item will need to be identified for the chart abstracter to easily locate (e.g., flowsheet, orders, MAR). Examples are provided in bold related to EPIC flowsheets.

**1.** Care Environnent<br>**Location of care (Inpatient vs. Outpatient)**	Critical Care: Neonatal Intensive Care (Level 4 or 3 NICU, ICN), Pediatric Intensive Care (PICU), Pediatric Cardiac Intensive Care (PCICU)<br>Intermediate Care: Transitional or Special Care Nursery, Neonatal Intensive Care (Level 2), Pediatric Bone Marrow Transfusion Unit, Pediatric Progressive Care, General Pediatrics<br>Outpatient (Complex): Patient still local for treatment, requiring frequent clinic visits, and have not been discharged 'home'<br>Outpatient: any patient not currently hospitalized and at home
**2.** Monitors<br>**Flowsheets → Vitals or Daily Cares**	Pulse oximeter includes continuous pulse ox monitoring and intermittent monitor if > 1 time/day<br>Cardiac-respiratory monitor includes continuous ECG monitoring<br>Blood pressure monitor includes continuous monitoring and intermittent monitoring if > 1 time/day<br>Pacemaker includes patients with pacemakers and those with pacemaker wires after cardiac surgery until the devices are removed, even if device is not pacing
**3.** Respiratory Assistance<br>**Flowsheets → Vitals or Daily Care**	RA: room air (21%), no respiratory support<br>NC with or without 02: >21% oxygen delivered via nasal cannula; includes high flow nasal cannula regardless of % oxygen delivered<br>CPAP or BIPAP: continuous positive airway pressure or Bidirectional positive airway pressure (i.e., RAM, Bubble CPAP, or another device)<br>Tracheotomy: tracheostomy present **will have additional score if on ventilator or other means of respiratory support.*<br>Ventilator: intubated (ETT) on a normal ventilator<br>High Frequency ventilator: intubated (ETT) on JET or oscillator ventilator
**4.** Invasive Lines<br>**Flowsheets → IV Assessment or "LDAs"**	UAC: umbilical arterial catheter<br>UVC: umbilical venous catheter<br>PAL: peripheral arterial line, also known as "A-lines"<br>PICC: peripheral inserted central catheters<br>PIV: peripheral inserted venous catheter<br>Central Line & Other: lines inserted to/ near heart (port-a-caths, hickmans, broviacs)
**5.** External Drains and/or Catheters<br>**Flowsheets → "LDAs" or assessment**	Includes all tubes and drains such as NG, OG, G-tube, Replogle, urinary catheters, chest tube, etc. Count present of any tube (1 point per chest tube).

**6.** Skin Care<br>**Flowsheets → NICU Body System → Skin Assessment**<br>- May need to check MAR for topical ointments	<u>Skin monitoring daily</u> includes patients requiring skin assessment or at high risk for skin breakdown; patients turned Q 2-4 hours<br><u>Topical applications</u> include topical medications listed on MAR<br><u>Routine line dressing changes</u> includes dressing changes occur per hospital policy (i.e., weekly)<br><u>Complex line dressing changes or surgical/trauma wounds:</u> dressing that require frequent changes because of oozing or bleeding or soiling or closed surgical wounds without complications and any trauma wounds that have occurred from care<br><u>Ostomy dressings and maintenance</u> include patients with ostomies<br><u>Open surgical wounds</u> include open wounds from surgery such as open chest or open abdomen
**7.** Oral Care (specialized)<br>**Flowsheets → NICU Body Systems → hygiene care**<br>- Check MAR for meds (e.g., Nystatin and mouth rinses)	<u>Routine:</u> mouth care that occurs daily or mouth care per hospital policy<br><u>Intermediate</u>: mouth care for patients with unrepaired cleft palate or patient receiving special medication for mouth care<br><u>Complex:</u> mouth care requiring multiple medication applications per day
**8.** Nutrition<br>**Flowsheets → I/O or heading about Feeding**<br>- Some patients may have both TPN & PO intake, depending on NPO status	<u>PO:</u> taking food by mouth, or chest feeding<br><u>Tube Feedings (gavage):</u> receiving food via tube (any type)<br><u>TPN:</u> receiving total parental nutrition and/or lipids
**9.** Blood Draws<br>**Review orders report for blood draws or results review (for labs)**<br>- Start on date of scoring, then count back 7 days and note any time there was a blood draw.<br>- Lab results would include Blood Gas, CBC, Basic Metabolic Panel (BMP), Cultures, etc.<br>- Do NOT count blood sugar check.<br>- Not counting the total # of blood draws, rather the daily frequency or presence of a blood draw.	<u>Daily:</u> at least 1x blood draw every day for 7 days<br><u>2/3 times a week</u>: blood draws between 2 – 6 days<br><u>Weekly:</u> once a week draws (1x in 7 days, even if multiple labs in 1 day)<br><u>None:</u> no blood draws in the previous 7 days
**10.** Colloid Therapy<br>**Flowsheets → Labs, Orders, or Transfusion Flowsheet**	Includes the total number of administrations of colloid products in the previous 7 days. Colloids include platelets, packed red blood cells, albumin, plasmanate, etc. Start on date of scoring, then count back 7 days and note number of transfusions in pervious 7 days.
**11.** Mobility<br>- Likely scored during outpatient via parent report or specialized therapy notes.	<u>Walkers:</u> patients requiring a walker to ambulate<br><u>Wheelchairs:</u> patients requiring wheelchair to be mobile<br><u>Special car seats:</u> special car seats (e.g., car bed). This does NOT include regular car seats for infants and children.

<table>
<tr><td></td><td colspan="2"><u>Special strollers:</u> strollers that are made for infants and children with disabilities. This does NOT include regular strollers</td></tr>
<tr><td rowspan="3">12. Medications
MAR or Orders
- Create additional boxes to correspond with total number of medications given on date of scoring (see example box below).
- Do NOT include medications already counted in TPN, topical skin care, or other domains.
- Weekly and Monthly administration are scored as a daily frequency.
- PRN medications are scored only if they were given on the day of scoring (e.g., Tylenol ordered PRN post-surgery, allocate points if given via route and frequency).
- Continuous maintenance infusions (e.g., D10 or infusions to keep lines open, KVO) are NOT scored (e.g., no points). For instance, an infant with a PIV and D10 for glucose instability would only have 1 point for the presence of the line and no score for maintenance fluids.</td><td>Frequency:</td><td><u>Daily:</u> administration of medication once per day (Q24), weekly, or monthly
<u>2-3x day:</u> administered multiple times a day (Q12, Q8), including PRNs
<u>4-6x day:</u> administered multiple times a day (Q6, Q4, Q2), including PRNs
<u>Continuous:</u> administered > 6 times per day or through continuous drip (e.g., dopamine, versed, fentanyl, etc)
<u>PRN:</u> only score medication if given on the day of scoring</td></tr>
<tr><td>Route:
IV, IM, ETT, Sub Q, Neb[6]</td><td><u>IV:</u> intravenous medications
<u>IM:</u> intramuscular medications
<u>Sub Q:</u> administration via subcutaneous tissue
<u>Neb:</u> inhalation medications delivered via a nebulizer
<u>ETT:</u> administration via endotracheal tube, includes surfactant</td></tr>
<tr><td>Route:
PO, Topical, Rectal, IN[7]</td><td><u>PO:</u> medication administered via mouth or NG, OG tube and includes all eye drops
<u>Topical:</u> medications ordered for topical administration (not Nystatin)
<u>Rectal:</u> medications ordered for suppository administration
<u>IN:</u> inhalation medications delivered via inhaler and includes all nasal sprays</td></tr>
<tr><td colspan="3">BIPAP: Bidirectional positive airway pressure, CPAP: Continuous positive airway pressure, ET: Endotracheal tube, GT: G-Tube, HFV: High frequency ventilation, IN: Inhalation, IV: Intravenous, IM: Intramuscular, NC: Nasal canula, Neb: Nebulizer, OG/ NG: Feeding Tubes, PO: Oral, or by mouth, Trach: Tracheostomy</td></tr>
</table>

Example medication box for scoring: *each medication scored individually (per box) and then summed.*

<table>
<tr><td colspan="5">Medication Name/Dose/Frequency:</td></tr>
<tr><td>Frequency</td><td>Daily</td><td>2-3 x/day</td><td>4-6x/day</td><td>Continuous</td></tr>
<tr><td>Route:
IV, IM, SubQ, Nebulizer, ETT</td><td>1 point</td><td>1.25 point</td><td>1.50 point</td><td>1.75 point</td></tr>
<tr><td>Route:
PO, Topical, Rectal, Inhalation</td><td>0.5 point</td><td>0.75 point</td><td>1 point</td><td>NA</td></tr>
</table>

**Table S1. TDI Rater Percent Agreement by Time (Study 1 & 2)**			
**Item**	**T1 - admission**	**Time 2 - inpatient**	**Time 3 - discharge**
1. Care Environment	100.0%	97.4%	97.5%
2. Monitors	100.0%	97.4%	97.5%
3. Parenteral Lines	97.5%	94.9%	95.0%
4. Respiratory Assistance	97.5%	94.9%	95.0%
5. Nutrition	100.0%	94.9%	72.5%
6. External Catheters or Drains	95.0%	87.2%	77.5%
7. Blood Draws	100.0%	97.4%	97.5%
8. Colloid Therapy	95.0%	92.3%	85.0%
9. Skin Care	87.5%	94.9%	100.0%
10. Oral Care	97.5%	94.0%	97.5%
11. Mobility	100.0%	100.0%	100.0%
12. Medication	47.5%	46.2%	45.0%

**Table S2. Interclass Correlations of Total Scores**			
	**T1 – admission**	**T2 – inpatient**	**T3 – discharge**
Both Studies	0.980	0.974	0.985
**Study 1:** Maternal Caregiving	0.958	0.966	0.993
**Study 2:** Cycled Light	0.904	0.977	0.962
